# Supplementary material for: Subcortical electrophysiological activity is detectable with high-density EEG source imaging
Source: Nat Commun. 2019 Feb 14;10:753. doi: 10.1038/s41467-019-08725-w (PMC6376013; doi:10.1038/s41467-019-08725-w)
Supplement: Supplementary file 1 — Supplementary Information [file 41467_2019_8725_MOESM1_ESM.pdf]

Subcortical electrophysiological activity is detectable with high-density EEG source imaging

Seeber et al.

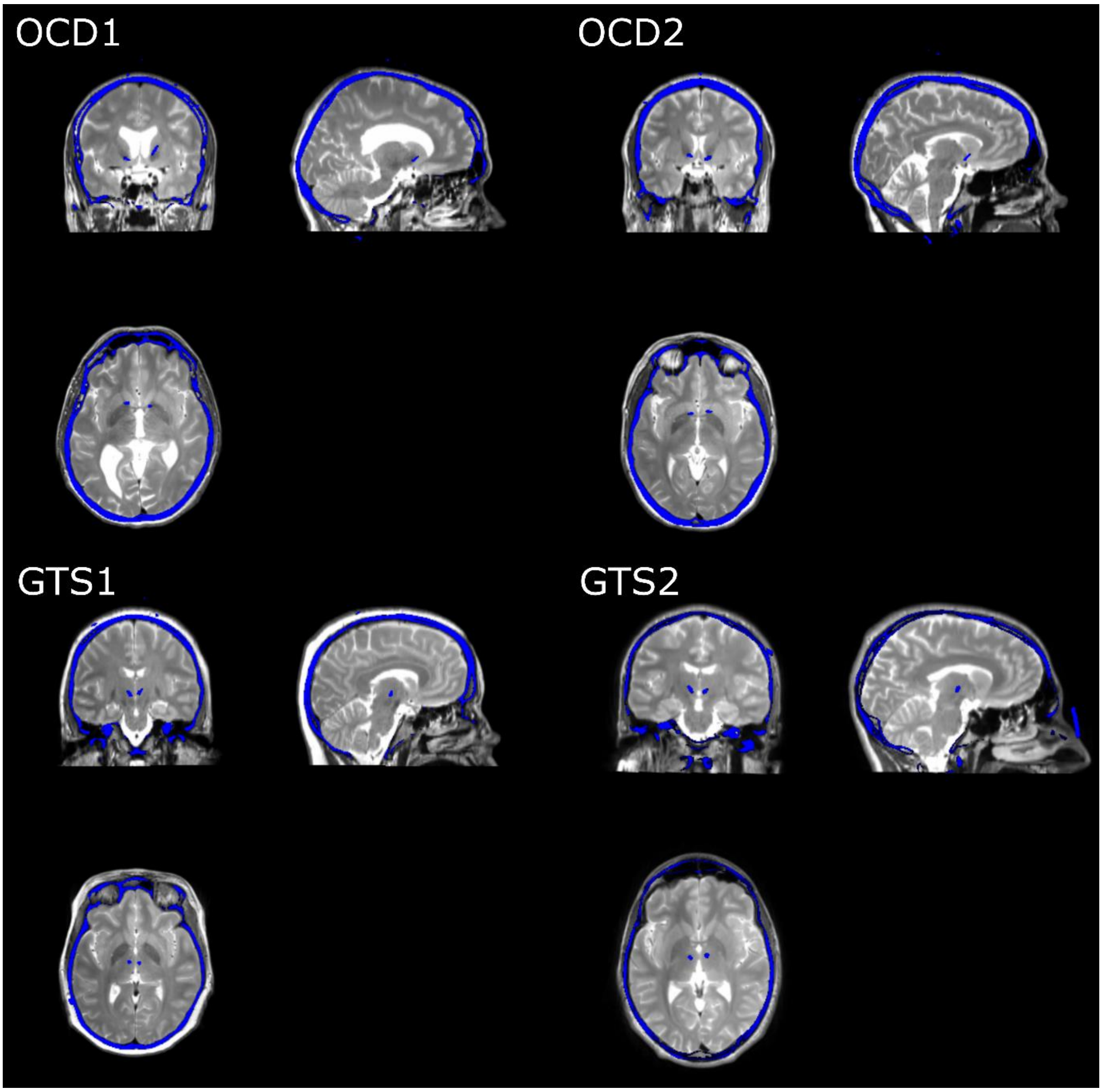

Supplementary Figure 1. Implanted electrode locations. T2 MRI (greyscale) overlaid with post-op CT (blue) scans, focusing on the DBS electrode locations. Electrode locations can be seen as blue dots showing the implantation site at the nucleus accumbens for OCD and centromedial thalamus for GTS patients.

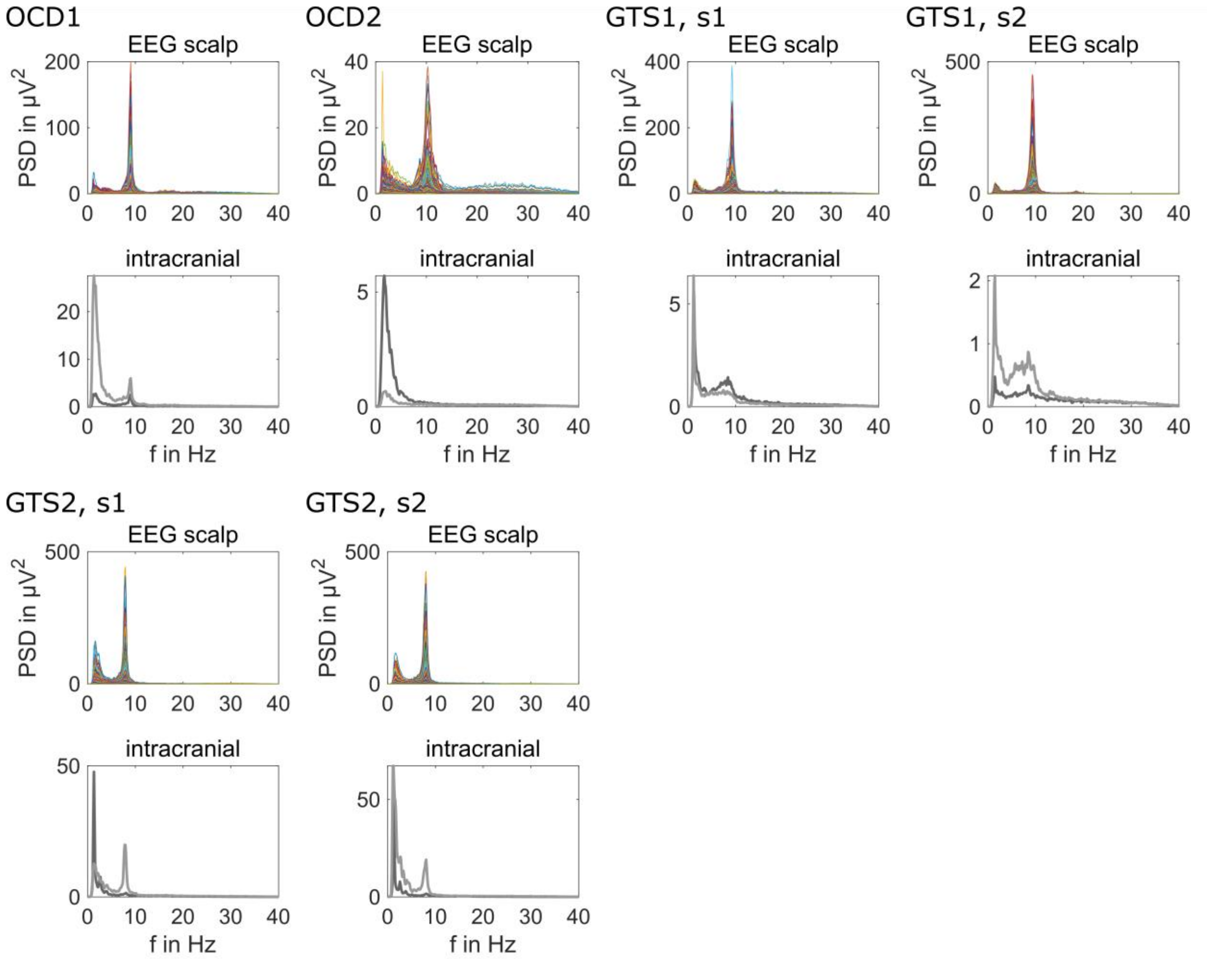

Supplementary Figure 2. Power spectral density plots of scalp EEG and intracranial recordings for every subject. Two different sessions were available for GTS1 and GTS2. Note that in OCD2 there was no alpha peak detectable and therefore excluded from further analysis. Light/dark grey colors correspond to left/right hemispheric implantation sites

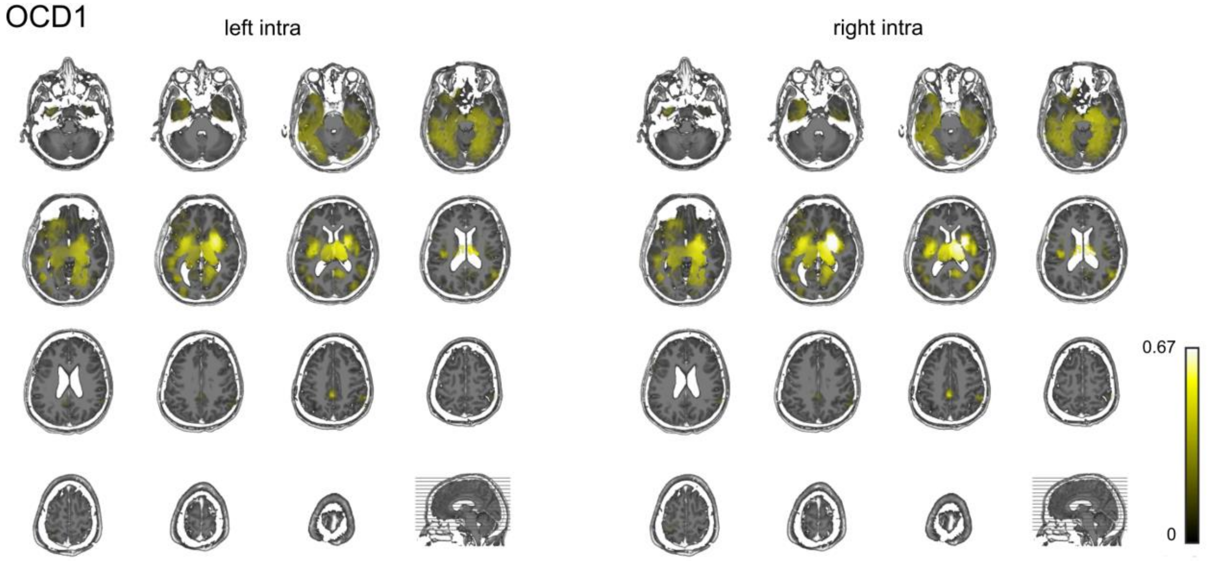

Supplementary Figure 3. EEG source images (OCD1) showing correlation between source reconstructed and actually recorded alpha envelopes derived from intracranial electrodes placed in the nucleus accumbens. Note, these images are displaying transversal slices of the whole brain and include non-significant correlations.

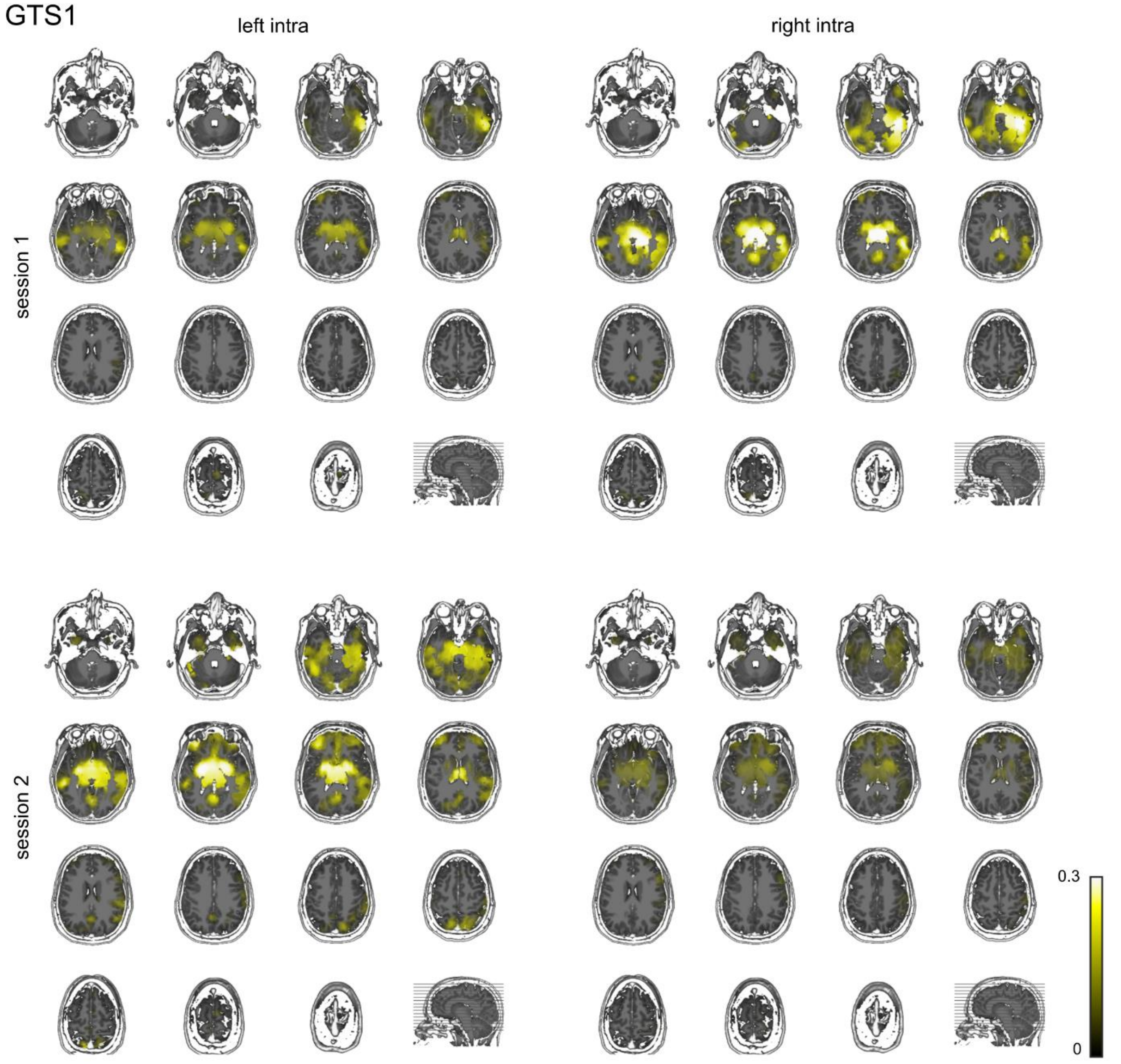

Supplementary Figure 4. EEG source images (GTS1) showing correlation between source reconstructed and actually recorded alpha envelopes derived from intracranial electrodes placed in the centromedial thalamus. Note, these images are displaying transversal slices of the whole brain and include non-significant correlations.

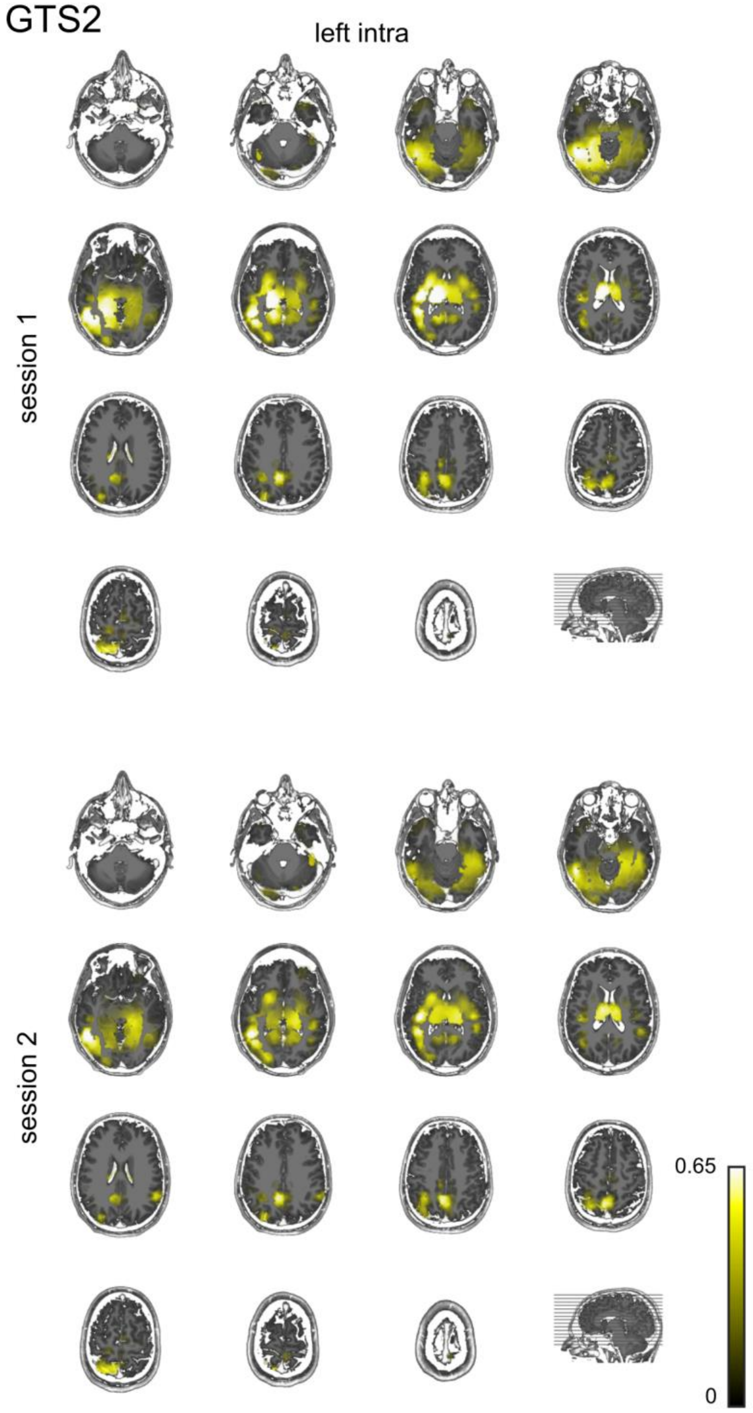

Supplementary Figure 5. EEG source images (GTS2) showing correlation between source reconstructed and actually recorded alpha envelopes derived from intracranial electrodes placed in the centromedial thalamus. Note, these images are displaying transversal slices of the whole brain and include non-significant correlations.
